# Interactive assistance via eHealth for small- and medium-sized enterprises’ employer and health care manager teams on tobacco control (eSMART-TC): protocol for a cluster randomized hybrid type II trial (N-EQUITY2101/J-SUPPORT2102)

**DOI:** 10.1186/s43058-023-00444-0

**Published:** 2023-06-07

**Authors:** Junko Saito, Miyuki Odawara, Maiko Fujimori, Aya Kuchiba, Shunsuke Oyamada, Khin Thet Swe, Eiko Saito, Kota Fukai, Masayuki Tatemichi, Masakazu Nakamura, Yosuke Uchitomi, Taichi Shimazu

**Affiliations:** 1 Division of Behavioral Sciences, National Cancer Center Institute for Cancer Control, Tokyo, Japan; 2 Division of Survivorship Research, National Cancer Center Institute for Cancer Control, Tokyo, Japan; 3 Innovation Center for Supportive, Palliative and Psychosocial Care, National Cancer Center Hospital, Tokyo, Japan; 4 Division of Biostatistical Research, Institute for Cancer Control/Biostatistics Division, CRAS, National Cancer Center, Tokyo, Japan; 5 Graduate School of Health Innovation, Kanagawa University of Human Services, Kanagawa, Japan; 6 Department of Biostatistics, JORTC Data Center, Tokyo, Japan; 7 Research Center for Health Policy and Economics, Hitotsubashi Institute for Advanced Study, Hitotsubashi University, Tokyo, Japan; 8 Institute for Global Health Policy Research, Bureau of International Health Cooperation, National Center for Global Health and Medicine, Tokyo, Japan; 9 Department of Preventive Medicine, Tokai University School of Medicine, Kanagawa, Japan; 10 Health Promotion Research Center, Institute of Community Medicine, Japan Association for Development of Community Medicine, Tokyo, Japan

**Keywords:** Employer, Health manager, Smoking cessation, Implementation, Small- and medium-sized enterprises, Workplace health promotion

## Abstract

**Background:**

Tobacco control should be a higher public health priority in Japan. Some workplaces provide smoking cessation support and connect employees to effective smoking cessation treatments such as outpatient clinics. However, tobacco control measures have not been sufficiently implemented in Japan, especially in small- and medium-sized enterprises (SMEs), where resources are limited. Organizational commitment and consistent leadership are crucial to facilitate implementation, but research on whether supporting organizational leaders leads to health behavior changes among employees is limited.

**Methods:**

This hybrid type II cluster randomized effectiveness implementation trial (eSMART-TC) aims to examine the effects of interactive assistance for SME management on health and implementation outcomes. We will provide interactive assistance to employers and health managers for 6 months, aiming to promote the utilization of reimbursed smoking cessation treatments by public health insurance and to implement smoke-free workplaces. The intervention will consist of three strategies: supporting employees through campaigns, tailored ongoing facilitation, and ensuring executive engagement and support. The primary health and implementation outcomes will be salivary cotinine-validated 7-day point-prevalence abstinence rate, and the adoption of two recommended measures (promoting utilization of smoking cessation treatment and implementing smoke-free workplaces) 6 months after the initial session, respectively. Other outcomes for implementation (e.g., penetration of smoking cessation clinic visits), health (e.g., salivary cotinine-validated 7-day point-prevalence abstinence rate at 12 months), and process (e.g., adherence and potential moderating factors) will be collected via questionnaires, interviews, logbooks, and interventionists’ notes at 6 and 12 months. An economic analysis will be undertaken to assess the cost-effectiveness of the implementation interventions at 12 months.

**Discussion:**

This will be the first cluster randomized controlled trial to evaluate the effectiveness of an implementation intervention with interactive assistance for employers and health managers in SMEs on smoking cessation and implementation of evidence-based tobacco control measures in SMEs. The findings of this trial targeting management in SMEs have the potential to accelerate the implementation of evidence-based smoking cessation methods as well as abstinence rates among employees in SMEs across Japan.

**Trial registration:**

The study protocol has been registered in the UMIN Clinical Trials Registry (UMIN-CTR; ID: UMIN000044526). Registered on 06/14/2021.

**Supplementary Information:**

The online version contains supplementary material available at 10.1186/s43058-023-00444-0.

Contributions to the literature

- This protocol for the first hybrid type II cluster randomized effectiveness implementation trial aims to examine the effects of interactive assistance to encourage behavioral change among employers and health managers.
- The implementation intervention will use the bundle of three implementation strategies (supporting employees through campaigns, tailored ongoing facilitation, and ensuring executive engagement and support) targeting employers and health managers.
- This study can help accelerate the implementation of evidence-based smoking cessation measures and increase the abstinence rate in Japan and the approach of this theory-informed multilevel intervention targeting management can be applied to other areas of the workplace.

## Background

Smoking is the most preventable risk factor for all-cause mortality in Japan. Although the overall smoking prevalence continues to decline over time, 16.7% of Japanese adults (27.1% men and 7.6% women), including 31.8–36.5% of men aged 30–50 years, were smokers in 2019 [1]. Additionally, owing to population aging and the pervasive health effects of smoking, the number of deaths per year due to smoking increased from approximately 129,000 in 2007 to approximately 190,000 in 2019 [2, 3].

In Japan, the “Standard Procedures for Smoking Cessation Treatment” at registered medical institutions has been provided as an evidence-based intervention under health insurance coverage since 2006. The reimbursed treatment program comprises five treatment sessions over a 12-week duration. Nicotine patches or varenicline could be prescribed during the treatment period. The abstinence rate was approximately 60% when the treatment session finished (including those who dropped out during the process) and approximately 27.3% 9 months after [4]. Despite being the most effective method for smoking cessation available in Japan [5], less than 20% of smokers who quit have used the treatment [6], mainly because of lack of access (only 15% of medical facilities offer smoking cessation treatments) or media campaigns to promote the use of such treatments [7].

The workplace is an ideal setting to reach people of diverse ages and sociodemographic statuses, with or without access to healthcare [8]. It offers a place to provide smoking cessation support and motivate employees to connect with more effective smoking cessation treatments, such as outpatient clinics [9]. Furthermore, a meta-analysis reported that a smoke-free policy in the workplace reduces not only secondhand smoke among employees, but also smoking prevalence by 3.8% [10]. Currently, tobacco control measures in the workplace have not been sufficiently implemented in Japan, especially in small- and medium-sized enterprises (SMEs) where resources are limited. For instance, according to a survey of SMEs in Japan, only 20% of enterprises were engaged in health promotion activities [11], and a national survey showed that the implementation rate of comprehensive smoke-free policies was lower than that of large companies [12].

Organizational commitment and consistent leadership from employers facilitate the implementation of health promotion interventions, including tobacco control measures [13]. Employers’ leadership in SMEs generally have a greater influence on employees than in large enterprises; thus, encouraging employers to become involved may be effective [14, 15]. However, most previous studies worldwide, including Japan, examined the effects of worksite interventions that directly deliver counseling and pharmacotherapy for smoking cessation to individual employees [9, 16, 17], and, to the best of our knowledge, only two worksite tobacco control programs in the USA examined whether providing interventions to organizational leaders (e.g., employers) leads to changes in managements’ implementation and employees’ health behaviors. The Working Well Trial, an intervention for cancer prevention including smoking cessation, supported worksite managements to plan and implement a smoking policy in addition to providing cessation support for employees [18]. Although there was no significant difference in the reduction in employee smoking prevalence compared to the control group, intervention sites were more likely to initiate and maintain mechanisms for institutionalized tobacco control programs. However, these do not focus on SMEs [19]. Health Links is an intervention supporting employers of SMEs to implement evidence-based interventions, including tobacco control, rather than directly supporting employees. Although the implementation score of tobacco control increased in intervention sites, the change of smoking prevalence among employees has not been examined [20, 21]. In addition, economic evaluations of such interventions have been quite limited, despite cost-effectiveness and budget impact being significant factors in decision-making for organizations [22].

Given these evidence gaps, rigorous trials are needed to examine the effects of interactive assistance for SME management for worksite tobacco control measures on health and implementation outcomes.

### Objectives

This hybrid type II cluster randomized effectiveness implementation trial aims to examine the effects of the interactive assistance via eHealth for SMEs’ employers and healthcare manager teams on tobacco control (eSMART-TC) on health and implementation outcomes.

#### Aim 1 (health outcomes)

To examine whether providing interactive assistance to encourage behavioral change among employers and health managers leads to a higher successful smoking cessation (the salivary cotinine-validated 7-day point-prevalence abstinence rate) at 6 months after the initial session compared to enterprises that did not provide interactive assistance.

#### Aim 2 (implementation outcomes)

To examine whether providing interactive assistance to encourage behavioral change among employers and health managers leads to a higher score of implementation of evidence-based tobacco control measures 6 months after the start of the intervention (control group provided with information) compared to enterprises that did not provide interactive assistance.

#### Aim 3 (cost)

To examine the cost-effectiveness of workplace smoking cessation as an incremental cost per quit in the intervention group versus the control group over the course of the intervention at 12 months after the initial session.

## Methods

### Study design and setting

This trial was designed as a parallel two-arm cluster randomized controlled trial, randomizing SMEs in a 1:1 ratio. A cluster design was selected because the intervention targeted employers, and the setting was the entire workplace. The definition of SMEs in this study includes an employee size of 30–300, based on the definition by the Small and Medium Enterprise Agency [23]. This protocol manuscript has been reported according to the Standard Protocol Items: Recommendations for Interventional Trials (SPIRIT) guideline checklist [24](supplementary file 1).

**Fig. 1:**
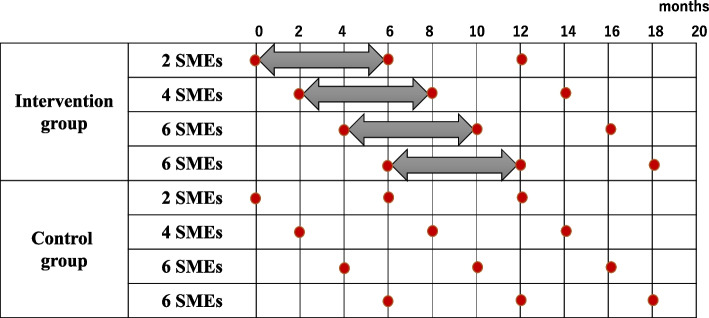
Data collection and intervention timeline. SMEs, small- and medium-sized enterprises ↔ refers to the intervention period, and red circles refer to data collection

As shown in Fig. 1, the outcomes will be measured 6 and 12 months after the intervention is implemented in an ongoing basis. Implementation of the 6-month intervention will be sequential as interventions are implemented by three members of the study team. The evaluation, including primary outcomes, will be conducted after the intervention across all sites is combined at 6 and 12 months.

### Study clusters

The study clusters will include SMEs belonging to the Japan Health Insurance Association (JHIA), the largest medical insurer in Japan covering approximately 2.5 million enterprises [25]. The JHIA has 47 branches covering all prefectures across Japan and each branch issues a “health declaration” certificate to enterprises actively working to promote employee health. Certified enterprises are required to appoint a health manager who plans and implements health promotion activities at their workplace. As this intervention will target enterprises with a high degree of readiness for health-promotion activities, we will recruit enterprises with health declarations. In case of multiple locations within a SME, the study cluster will cover only the headquarters and branches on the premises of that location. This is because we assume that the effects of health promotion activities may be smaller for employees in more distant locations compared to those in closer locations within the same or walking distance.

Inclusion criteria for SMEs:

1. In business for more than 3 years.
2. An SME or a branch or business office of an enterprise, with 30–300 employees.
3. Have already entered the Health Declaration conducted by a branch of the JHIA.
4. Both the employer and health manager are aged ≥ 20 years.
5. Facilities for conducting web-based sessions are available in the workplace.
6. The employer’s consent for participation has been obtained.
7. High level of readiness to implement tobacco control measures.

A high level of readiness is defined as follows:

- The employer is available to participate in sessions: 2–3 sessions (30 min to an hour) during the first 2 months of the intervention.
- The health manager is available to participate in sessions: once a month for the first 3 months of the intervention and twice a month from the fourth to the sixth month.
- Health managers can set aside time to implement the measures.

### Study population

The study population will include intervention and evaluation targets. The intervention targets will be an employer and a health manager at each participating SME. In cases where it is difficult for an employer to participate, it will be possible for a person in a managerial position to implement measures within the enterprise to participate in the study as the equivalent of an employer.

The evaluation target will include all employees of the participating SMEs, in addition to the intervention targets. Thus, for employee-level analysis, all employees of randomized enterprises who meet the eligibility criteria will be included in the analysis.

Inclusion criteria for employers:

1. An employer or someone with influence on decision-making at eligible SMEs.
2. Aged ≥ 20 years.
3. Able to read, write, and understand Japanese.

Inclusion criteria for health managers:

1. Involved in fostering health management and promotion among employees at eligible SMEs.
2. Aged ≥ 20 years.
3. Able to read, write, and understand Japanese.

Exclusion criteria for employers and health managers:

1. Those who have already decided to move or change jobs within the next year and plan to leave their current affiliation.
2. Those who are deemed unsuitable by the JHIA branch office for participating in this study.

### Recruitment

The following procedures will be used to register the SMEs for this study: first, we will recruit JHIA branches that agree to cooperate with SMEs’ recruitment by consulting with the headquarters of the JHIA. The recruitment will be conducted sequentially, starting with the branches that have come forward and continuing to recruit additional branches until the target of 36 SMEs has been recruited. Second, we will ask each branch that has offered to cooperate to prepare a list of SMEs that meet the inclusion criteria and mail flyers outlining the study. Third, the research team will organize recruitment briefings for potential SMEs that have responded with interest in this study to clarify the study’s overview and answer any questions they may have. Finally, the employer and health manager of the SMEs that decide to participate will provide written informed consent before the study begins (Supplementary file 2).

### Random allocation and adjustment factors for allocation

Each enterprise will be randomly assigned in equal proportion to the intervention or control group using a stratified randomization method of static allocation with two adjusted allocation factors at the workplace level: number of employees (less than 100, 100 or more) and implementation of a comprehensive smoke-free policy on the workplace (yes or no). All employees eligible for the evaluation target (i.e., employees working at the headquarters and branch on the premises of that location) will be allocated to the same group. The workplaces will be randomly allocated by an independent research assistant not involved in the study using computer-generated random numbers prepared in advance by a statistician.

Owing to the nature of the intervention, it will not be possible to blind the assignment of the participants (employers and health managers) and interventionists. They will be informed of their assignment after the baseline assessment is completed. However, the assessor of the biochemical salivary test will be blinded to the randomization groups; the study statistician will also be blinded to the randomization groups until the analysis of primary outcomes at 6-month follow-up is conducted.

### Intervention

The intervention will be provided for employers and health managers in each of the participating enterprises over 6 months. Interventionists will be three research team members (MO, TS, and JS) specializing in psychology, medicine, and nursing, respectively. The team member who specializes in psychology will train the other two interveners in advance using the intervener manual. The manual provides examples of dialogs with employers and health managers, as well as various combinations of behavior modification approaches that should be offered for each factor.

**Fig. 2:**
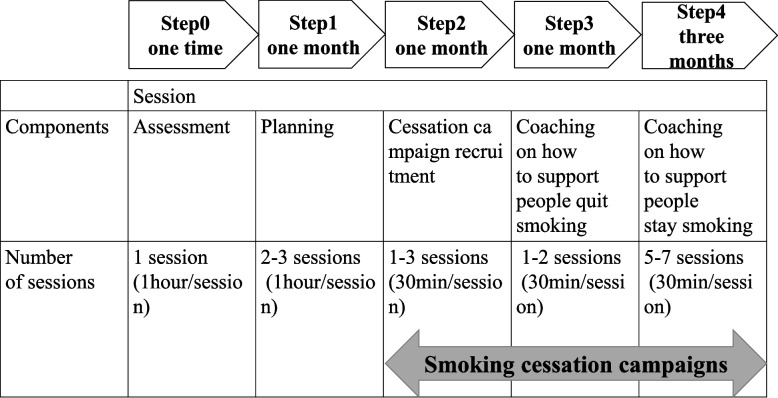
Steps and components of the intervention and number of sessions

As shown in Table 1, the intervention will consist of a bundle of three implementation strategies (supporting employees through campaigns, tailored ongoing facilitation, and ensuring executive engagement and support), developed based on our prior work, which demonstrated that strong endorsement, support, and positive feedback from employers are important to promote the implementation of workplace health promotion [13]. It also identifies strategies for the prevention of non-communicable diseases for SMEs in Japan following an implementation mapping protocol [26]. These three implementation strategies will be provided through five steps in web-based sessions, with four sessions for employers and ten sessions for health managers (maximum of 16) (Fig. 2). In principle, the sessions will be conducted online using a web conferencing system but may be conducted in person if the situation warrants. From 1 month after the intervention begins, each enterprise will be required to start the smoking cessation campaign with an option of the incentives for successful quitters. As multi-level interventions are more effective, if a complete smoke-free policy is not in place, it is recommended that they are implemented at the same time as, or around the time of, the launch of the campaign.

**Table 1:** Implementation strategies

Implementation strategy	Description	ERIC category	Description according to Proctor [27]	
Supporting employees to quit smoking through the cessation campaign	Ongoing support for smoking cessation campaigns in the workplace, from planning to implementing and evaluating the campaign. The duration of the smoking cessation campaign, the definition of successful quitters, the option of incentives for quitters, and how the campaign is communicated are discussed and supported together with the health manager to ensure that the content is best suited to the characteristics of the workplace	Develop educational materials Distribute educational materials Conduct ongoing training	Actor:	Interventionist who is an expert in smoking cessation support
			Action:	Provide periodic interviews with educational materials according to the predefined schedule during the 6 months
			Targets of the action:	Employers and health managers
			Temporality:	The aim and objective of the campaign at the workplace should be set within the first month of the program; The leadership declaration should be made before the smoking cessation campaign begins
			Dose:	Intensive communication in the first 1 month interview; ongoing support throughout the intervention period
Ongoing facilitation tailored to the workplace context	An interactive support through regular sessions with employers and health managers, including feedback and problem-solving tailored to the workplace situation, in order to improve their fidelity to implement tobacco control measures. Information for facilitation can be obtained from the initial assessment session as well as from action memos describing the latest smoking or cessation status of declared employees, support provided to them, and their reaction to the support, which will be submitted to interventionist before each session	Audit and provide feedback Facilitation Tailor strategies	Actor:	Interventionist who is an expert in smoking cessation support
			Action:	Provide ongoing support in a companionate manner by presenting solutions to the problems and challenges faced by each company at any given time
			Targets of the action:	Employers and health managers
			Temporality:	Every regular interview throughout the intervention period
			Dose:	Every regular interview and optional support when needed via e-mail and phone in addition to online meeting throughout the intervention period
Ensuring executive engagement and support	Executive engagement includes set the purpose of the smoking cessation campaign at the workplace, short and long goals with performance measures, and timeframe. Obtain formal commitments from executives to declare the priority of the cessation campaign as well as purpose and aim in front of all employees	Develop a formal implementation blueprint Mandate change	Actor:	Interventionist who is an expert in smoking cessation support
			Action:	Provide information on precedents where tobacco control programs have been successfully developed with appropriate goal setting and will facilitate goal setting
			Targets of the action:	Employers
			Temporality:	The aim and objective of the campaign at the workplace should be set within the first month of the program; The leadership declaration should be made before the smoking cessation campaign begins
			Dose:	Intensive communication in the first 1 month interview; ongoing communication of support and endorsement throughout the intervention period

*ERIC* Expert Recommendations for Implementing Change [28]

In Step 0 (assessment), interventionists will assess the barriers that employers and health managers are facing for the implementation of tobacco control measures, using a workplace tobacco control checklist. In Step 1 (planning), interventionists will provide feedback on the results of the workplace assessment and support the development of a plan to implement tobacco control measures, including setting the purpose of implementing tobacco control measures, short- and long-term goals, and the timing for achieving them. Furthermore, interventionists will encourage employers to declare their set objectives and goals directly in front of all employees. In Step 2 (cessation campaign recruitment), interventionists will support the employers and health managers in recruiting smokers who are willing to quit and ask them to declare their willingness to quit. In Step 3 (supporting cessation initiation), interventionists will support the health manager to effectively help declared employees to start quitting by encouraging them to visit a smoking cessation clinic rather than quitting on their own. In Step 4 (supporting long-term cessation), interventionists will support the health manager to effectively help declared employees to continue to quitting, including the challenge of quitting again after a relapse. Health managers should speak with smokers who declared to stop weekly in principle and inquire about their smoking cessation status.

In Steps 1 and 2, a health manager will be asked to give top priority to recommending the first visit to the outpatient smoking cessation clinic and support the completion of the five visits. The next option will be to encourage the use of over-the-counter smoking cessation medications for smokers who choose not to visit outpatient smoking cessation clinics. Smokers who choose to quit smoking on their own and those who do not wish to use over-the-counter smoking cessation medications will be encouraged to quit smoking through regular encouragement by a health manager or colleagues in the same SME.

In our previous feasibility study, we confirmed that this web-based interactive intervention is feasible and acceptable for employers and health managers at SMEs in Japan. To ensure that having support from the enterprise does not put pressure on smokers, interventionists will repeatedly inform the employers and health managers that the enterprises must only support employees who are interested in quitting, never to force them to quit, and remember that smoking is an addiction that is difficult to quit solely based on their own willingness.

### Control group

The interventionist will provide the control group feedback on a summary of the results of the baseline survey (employee and workplace questionnaire) and information on existing materials on evidence-based smoking cessation measures (i.e., promoting utilization of smoking cessation treatment and implementing smoke-free workplaces). The timing of the feedback provision will be aligned with each completed employee questionnaire.

As a usual practice, both the intervention and control groups will receive workplace health promotion support and individual health guidance for targeted employees from health nurses in the JHIA.

### Outcomes

We will evaluate the health-, implementation-, process-, and cost-related outcomes. Outcome measures, evaluation period, and data sources are summarized in Table 2.

**Table 2:** Outcome measures by period and data source

	Baseline	Follow-up		
Outcome measures		6 months	12 months	Data source
Health outcomes				
Smoking status	〇	〇	〇	Employee questionnaire
Cotinine-validated 7-day point-prevalence abstinence rate		〇	〇	Employee questionnaire Biochemical test
30-day point-prevalence of smoking abstinence		〇	〇	Employee questionnaire
Three-month point-prevalence abstinence rate		〇	〇	Employee questionnaire
Six-month point-prevalence abstinence rate			〇	Employee questionnaire
Three months prolonged abstinence rate		〇	〇	Employee questionnaire
Six months prolonged abstinence rate			〇	Employee questionnaire
Proportion of nicotine patches and gum use		〇	〇	Employee questionnaire
Proportion of quit attempts	〇	〇	〇	Employee questionnaire
Number of cigarettes or capsules	〇	〇	〇	Employee questionnaire
Implementation outcomes				
Adoption of two recommended measures	〇	〇	〇	Workplace questionnaire
Penetration of smoking cessation clinic visits		〇	〇	Employee questionnaire
Process outcomes				
Adherence (content) (e.g., Was each recommended measure implemented as planned?)	●	●	●	Workplace tobacco control checklist Interventionists’ notes
Adherence (frequency/duration) (e.g., Were the recommended measures implemented as often and for as long as planned?)		●	●	Health manager’s logbooks Interventionists’ notes
Adherence (coverage) (e.g., What proportion of employees who smoke participated in the cessation campaigns?)		●	●	Health manager’s logbooks Interventionists’ notes
Potential moderators (responsiveness) (e.g., How did employees who smoke engage with the smoking cessation campaigns?)		●	●	Semi-structures interviews Interventionists’ notes
Potential moderators (complexity) (e.g., How complex is the intervention?)		●	●	Semi-structures interviews Interventionists’ notes
Potential moderators (comprehensiveness) (e.g., How specific is the interventions description?)		●	●	Semi-structures interviews Interventionists’ notes
Potential moderators (strategies) (e.g., What strategies were used to support implementation?)		●	●	Semi-structures interviews Health manager’s logbooks Interventionists’ notes
Potential moderators (quality) (e.g., How was the quality of the delivery of intervention components?)		●	●	Semi-structures interviews Health manager’s logbooks Interventionists’ notes
Potential moderators (recruitment) (e.g., What constituted barriers to maintaining involvement of employees?)		●	●	Semi-structures interviews Health manager’s logbooks Interventionists’ notes
Potential moderators (context) (e.g., What factors at political, economic, organizational, and work group levels affected the implementation?)		●	●	Semi-structures interviews Health manager’s logbooks Interventionists’ notes
Cost outcomes^a^				
Employees’ direct medical costs		〇	〇	Health Insurance database
The mean values and response rates for basic health check-up items		〇	〇	Health Insurance database
Workers’ lost productivity (absenteeism, presenteeism)	〇	〇	〇	Employee questionnaire
Cost of implementing tobacco control measures		〇	〇	Health manager’s logbooks

Black circles refer to collecting data for the intervention group only, and white circles refer to data collection for both groups

^a^Cost outcome measures will be collected throughout the year from baseline to 12 months

#### Health outcomes

The primary health outcome will be the salivary cotinine-validated 7-day point-prevalence abstinence rate 6 months after the initial session, according to the Russell Standard [29]. Abstinence rate will be assessed by self-reporting using an employee questionnaire with the following questions: “Have you smoked even one mouthful of tobacco [including all cigarettes, (cigarettes rolled in paper, heated, and other types)] in the last seven days?” Seven-day point-prevalence is defined as salivary nicotine-confirmed self-reported tobacco cessation in the last 7 days (response of “no” to the above question), and a cotinine concentration of 15 ng/ml or less will verify self-reported smoking abstinence [30]. A Smokerlyzer will be used to measure expired air carbon monoxide concentrations, with a cut-off point of six parts per million if self-reported quitters report using a nicotine patch or gum within 7 days of the salivary cotinine measurement date [31]. If an employee’s self-reported smoking abstinence does not match the biochemical measurement, the employee will be classified as a smoker.

Other health outcomes include the salivary cotinine-validated 7-day point-prevalence abstinence rate 12 months after the initial session, 30-day point-prevalence abstinence rate at 6 and 12 months, 3-month point-prevalence abstinence rate at 6 and 12 months, 6-month point-prevalence of abstinence rate at 12 months, the proportion of quit attempts at 6 and 12 months, and the number of cigarettes or capsules at 6 and 12 months among employees who report being smokers at the baseline survey. Among employees who smoke and declare to quit during the intervention period in the intervention group, the 3 months prolonged abstinence rate at 6 and 12 months, and 6 months prolonged abstinence rate at 12 months will be measured. In addition, we will assess the proportion of nicotine patches and gum use in the past 6 and 12 months measured at 6- and 12-month follow-ups, respectively, among employees who smoke at baseline.

Furthermore, the mean values and response rates for basic health check-up items (medical interview sheet, physical measurements, blood pressure, blood lipids, liver function, blood glucose, and urine) in all employees or in the smoking population will be obtained from the JHIA headquarters after each SMEs’ employer agrees.

#### Implementation outcomes

Implementation outcome is defined as “the effects of deliberate and purposive actions to implement new treatments, practices, and services,” and serves as indicators of the implementation success, showing progress in implementation processes, and as key intermediate outcomes related to health outcomes [32]. The primary implementation outcome in this study will be the adoption of two recommended measures (promoting utilization of smoking cessation treatment and implementing smoke-free workplaces) at 6 months (binary outcome) obtained from the workplace questionnaire that collects data at the worksite level.

Other implementation outcomes will include the adoption of two recommended measures at 12 months, and the penetration of smoking cessation clinic visits during the past 6 and 12 months among employees who report smoking at baseline, which will be evaluated using self-report responses from employee questionnaires at 6 or 12 months. The penetration is defined as “the number of eligible persons who use a service, divided by the total number of persons eligible for the service [32],” and could also be called in this study as the proportion of those who visited a smoking cessation clinic at least once during the intervention period among employees who smoked at baseline.

#### Process outcomes

For process outcomes, we will apply the modified conceptual framework for implementation fidelity by Hasson to select the process evaluation of the fidelity of implementation strategies that the employer and health manager are required to implement during the intervention: adherence and potential moderators [33]. Table 2 presents the components addressed: adherence (e.g., content and frequency or duration) and potential moderating factors (e.g., participant responsiveness, intervention complexity, comprehensiveness of policy description, and strategies to facilitate implementation). We will assess these components only in the intervention groups.

#### Cost-related outcomes

The cost-effectiveness of workplace smoking cessation will be estimated as the incremental cost per quit in the intervention group versus the control group over the intervention and follow-up periods (12 months after the initial session). The cost component accounts for three categories: (1) the cost of implementing tobacco control measures in the workplace, (2) employees’ direct medical costs during the study period, and (3) workers’ lost productivity.

The cost of implementing the measures includes the amount of time spent by employers and health managers on implementing the measures, converted into labor costs using the average salary level for the relevant age group in Japan, as well as cost for incentives, including cost assistance schemes for smoking cessation treatments, and the implementation of smoke-free policies. Employees’ direct medical costs consist of medical treatment costs (calculated from the total number of points in the monthly medical fee schedule) and medical expenses (calculated from the monthly total of the medical remuneration statement). Workers’ lost productivity will be estimated based on absenteeism and presenteeism. Absenteeism is the loss of productivity due to absence from work; the amount of labor cost loss will be calculated by converting the number of days of absence from work owing to sickness (from the questionnaire) into labor costs at the average salary level for the relevant age group in Japan. Presenteeism refers to the loss of productivity at work owing to an illness and performing at lower levels than usual. The amount of labor cost loss will be calculated by converting relative presenteeism [i.e., a ratio of actual performance to the performance of most workers at the same job (possible performance) from the World Health Organization Health and Work Performance Questionnaire, Japanese edition] into labor costs at the average salary level for the relevant age group in Japan.

### Data collection procedures

A multi-method approach will be used to collect data. Data collection methods will include questionnaires (employee questionnaire, workplace questionnaire, and workplace tobacco control checklist), biochemical tests, semi-structured interviews, health managers’ logbooks, interventionists’ notes, and cost-related data from health insurance database.

#### Questionnaire

Data from the employee questionnaires will be collected electronically using the electronic data capture system ViedocMe (Viedoc Technologies, Sweden) established by the data center. The QR code of the URL of this survey form will be printed and distributed to each employee, along with their individual account ID and password. During the predefined questionnaire response period, employees will log in using their individual account ID and password on the web page or by using an application on a smartphone or tablet device and then complete the survey. Once logged in, a check box will ask whether the participants agree to complete the survey, after an introduction to this project and the survey. If an employee selects the “Disagree” box, the survey form will be closed. If enterprises desire, printed paper responses will also be accepted. Saliva will be collected by mail from employees who report they have not smoked in the past 7 days at the 6- and 12-month follow-up [34]. Data from the workplace questionnaire and workplace tobacco control checklist will be collected electronically using survey applications, such as Google Forms and Microsoft Forms.

#### Biochemical test

For saliva collection, employees will receive a kit with instructions from a health manager at the workplace and provide verbal consent via telephone to the research staff prior to saliva collection. Samples returned to the research team will be stored in a refrigerator until they are mailed to an independent laboratory specializing in the analysis of biological samples. For employees who report using a nicotine patch or gum within 7 days of the salivary cotinine measurement date, we will ask them to measure expired air CO concentrations using a Smokerlyzer (Harada Industry, Osaka, Japan) online and show the results to the research staff through a screen.

#### Semi-structured interviews

Semi-structured interviews will be conducted only for intervention groups. The interviews with the employer and health managers will be conducted together in the same place at 6 months, while interviews with employees at 6 months will be conducted separately. Semi-structured interviews at 12 months will be scheduled for health managers only but will be conducted optionally if the results of the interview suggest that additional information is required from employers or employees. An interview guide will be developed with open-ended questions. For employers and health managers, the guide will cover acceptance and appropriateness of this intervention, the fidelity of tobacco control activities recommended during the intervention, and also covers the process of normalization of tobacco control measures in the worksite based on the Normalization Process Theory [35]. The guide will cover responsiveness, acceptance, and perceived context (all within potential moderators in the fidelity model) for employees. Each interview will last approximately 30–60 min. Interviews and focus groups will be conducted in Japanese, audio recorded, transcribed, and verified for accuracy. The interviewees will receive a coupon card (Quocard) for JPY 1000 as a reward.

#### Other data sources

Health managers will fill their logbooks in a timely manner whenever they take any action regarding tobacco control measures during the intervention and follow-up period, in both intervention and control groups.

#### Cost-related data

Health managers’ logbooks will be used to estimate the costs of implementing tobacco control measures in the workplace. For data on the direct medical costs of employees during the study period, the mean values for all employees or the smoking population in each SME will be obtained from the health insurance database at JHIA headquarters after obtaining each SMEs’ employer agreement. Finally, an employee questionnaire will be used to assess workers’ lost productivity. All data will be stored securely and will only be accessible to the research team and data manager.

### Sample size and power calculations

The purpose of this study is to test the effectiveness of an intervention that can be disseminated to small- and medium-sized enterprises with limited resources. To achieve this goal, we considered the intervention to be effective if it produces an abstinence rate that is equal to or greater than that in a previous study on similar interventions in a Japanese workplace setting [16]. Thus, we assumed the 7-day point-prevalence abstinence rate to be 13.3% for the intervention group 6 months after the intervention begins.

The smoking cessation percentage of the control group was estimated to be 3.7%. As a breakdown of the 3.7%, the 2.3% under natural behavior change was based on the difference in the average smoking rates between fiscal year 2018 and 2019 for companies belonging to the JHIA (only among companies with 10–999 employees), and the 1.4% as an impact of the revision of the Health Promotion Act was based on the proportion of enterprises that said they would promote tobacco control measures if the Health Promotion Act was revised, according to a survey of approximately 6,000 enterprises nationwide [36]. Thus, we assumed the abstinence rate in the control group would be higher than the 2.5% in the control group who received only information in the previous intervention study. [16]

With a mean of 15 smokers per cluster (assuming 50 employees and a smoking proportion of 30% in each SME) and an intra­class correlation of 0.05 based on a previous cluster randomized study in the workplace [37], which provides a design effect of 1.70, a sample size of 240 smoking employees within 16 clusters per group will be required with a two-sided significance level of 5% and 80% power. Given a 10% loss to follow-up due to unexpected employee turnover, resignation, or retirement, the planned sample size is at least 264 smokers in 18 clusters per group.

### Statistical analysis

The primary analysis for the primary outcome will be conducted on an intention-to-treat basis, that is all randomized participants who respond as being current smokers at baseline and have the biochemically confirmed 7-day point-prevalence abstinence data at 6 months will be included in the analysis and will be analyzed according to the group to which they were allocated. The primary analysis will exclude the participants lost to follow-up and no imputation of missing data will be performed; however, imputing missing data may be considered to account for all randomized participants in the sensitivity analysis. For the primary outcome, the biochemically confirmed 7-day point-prevalence abstinence rate at 6 months will be compared between the intervention and control groups, and the difference in the abstinence rates with 95% confidence intervals (CIs) will be estimated. A generalized estimating equation (GEEs) method with an identity link and an exchangeable correlation structure will be used. Robust variance will be used for hypothesis testing and estimation. The allocation factors of the number of employees and the implementation of a comprehensive smoke-free policy at the workplace will be adjusted in the GEE model. A two-sided *P* value of 0.05 or less will be considered statistically significant. Because of model convergence issues, we may also consider a logistic regression model with GEEs and an exchangeable correlation structure for hypothesis testing and estimating odds ratios with 95% CIs. Secondary outcomes, including implementation, and process, health, and cost-related outcomes, will also be analyzed. Detailed methods for the secondary outcomes and sensitivity analyses will be specified in the Statistical Analysis Plan before the study data are fixed.

Regarding adherence content, which will be measured in the intervention group only, we will conduct paired t-tests to examine pre- versus post-intervention changes in responses of the adherence measured by the workplace tobacco control checklist.

The incremental cost per quit will be estimated as the cost per smoker in the intervention group minus the cost per smoker in the control group divided by the incremental quit between the intervention and control groups. Probabilistic and deterministic sensitivity analyses will be performed to quantify the uncertainties associated with the input parameters and to assess the robustness of the base case. The deterministic analysis will assume a percentage variation of respective cost parameters from the base case. Monte Carlo simulation will be applied to conduct probabilistic sensitivity analysis by varying the input parameters based on the appropriate distributions.

### Content analysis

Qualitative data (logbooks, semi-structured interviews, and interventionists’ notes) will be analyzed using content analysis. As has been suggested [38], a coding scheme will be created and tested prior to the analyses and will be analyzed using both descriptive and analytical methods. For instance, the qualitative results of potential moderators will be compared using the subsequent component in the fidelity model, such as adherence to tobacco control activities at SEMs or the abstinence rate.

## Discussion

To the best of our knowledge, the eSMART-TC will be the first cluster randomized control study to apply a hybrid type II design to evaluate the bundle of three implementation strategies (supporting employees through campaigns, tailored ongoing facilitation, and ensuring executive engagement and support) targeting employers and health managers. This study will build upon our previous work, which demonstrated that strong endorsement, support, and positive feedback from employers are important to promote the implementation of workplace health promotion [13], and identified strategies for SMEs in Japan following an implementation mapping protocol [26]. This study will allow us to determine whether interactive intervention for management can increase the implementation of tobacco control measures in SMEs where resources are generally limited and increase the uptake of evidence-based cessation methods (smoking cessation treatments, nicotine replacement therapy, and nicotine gum) among smoking employees.

In the current implementation intervention, the total time spent by employers and health managers will be relatively long, ranging from 4–6 h (session participation time) for employers and 16.5–19 h (6.5–9 h interview participation time + 10 h implementation time) for health managers over the 6-month intervention period. A mixed-method approach to evaluate the process and implementation outcomes will allow us to identify the core components of the intervention. Consequently, the intervention is expected to be shorter, without compromising its effectiveness.

A key strength of our study is that the content of the implementation intervention was developed based on a theoretical framework and formative research. The implementation strategies were developed by applying social cognitive theory, targeting barriers and facilitators identified by the Consolidated Framework for Implementation Research [13, 26]. If the effectiveness of this intervention is confirmed, the approach of this intervention targeting management can be applied to other areas of the workplace, such as measures against hypertension and improving health check-up rates. Furthermore, the majority of a country’s workforce works in SMEs; for example, approximately 70% of Japanese employees and 50% of American employees work in SMEs [39] and face common challenges in implementing health promotion activities owing to lack of resources [40, 41]. If this implementation intervention for SMEs is adopted worldwide, its impact on the health, quality of life, and productivity of employees will be significant.

Our study may have two limitations. First, generalizability may be limited, as the characteristics of providers (i.e., employers and health managers) and the workplace (e.g., cultures, readiness to implement tobacco control measures, and industry type) might affect the adherence of health managers to implement the worksite tobacco control measures and the participation rate of employees in the campaign. Second, workplace assignment cannot be blinded because of the nature of the intervention. This might lead to disappointment or loss of motivation for employers and health managers in the control group to continue participating in this trial, as they need to wait 1 year to start the program.

Overall, our study will be highly meaningful as it has the potential to accelerate the implementation of evidence-based smoking cessation methods and increase the abstinence rate in SMEs across Japan by supporting employers and health managers to encourage and connect smoking employees to evidence-based cessation treatments to support the smoking employees directly.

### Trial status

Workplace recruitment began in July 2021 and this study is ongoing. At the time of the first submission in April 2023, an evaluation for 12 months is currently being conducted sequentially. Data analysis, including data cleaning, has not yet begun.

## Supplementary Information

**Additional file 1.** SPIRIT checklist.

**Additional file 2.** Letter of consent.

## Data Availability

Not applicable.

## Abbreviations

SME: Small- and medium-sized enterprise
JHIA: Japan Health Insurance Association
CO: Carbon monoxide
GEE: Generalized estimating equations
NCD: Non-communicable diseases
CI: Confidence interval
