# Supplementary material for: Interactive assistance via eHealth for small- and medium-sized enterprises’ employer and health care manager teams on tobacco control (eSMART-TC): protocol for a cluster randomized hybrid type II trial (N-EQUITY2101/J-SUPPORT2102)
Source: Implement Sci Commun. 2023 Jun 7;4:61. doi: 10.1186/s43058-023-00444-0 (PMC10249242; doi:10.1186/s43058-023-00444-0)
Supplement: Supplementary file 2 — Additional file 2. Letter of consent. [file 43058_2023_444_MOESM2_ESM.docx]

Letter of Consent

To the President of the National Cancer Center,

Interactive assistance via eHealth for small and medium-sized enterprises’ employer and health care manager teams on tobacco control

1. Purpose and significance of the study.

2. Establishment and target population of the study.

3. Content and methods of the study:

Group 1: □ Pre- and post-survey using questionnaires □ Interactive support intervention

Group 2: □ Pre- and post-survey using questionnaires □ Waiting and post-waiting interventions for interactive support

4. Anticipating the benefits and disadvantages of participating in this study.

5. Response and compensation in the event of health problems.

6. Participants will not be at a disadvantage if they do not participate.

7. Participants can withdraw their consent at any time after providing it.

8. Financial burden of participating in the research

9. Protection of participants’ privacy and personal human rights.

10. Publication of results and disclosure of information related to this research.

11. Secondary use of the data.

12. Ethical review of the study.

13. The period of participation, overall expected duration of the study, and expected number of participants.

14. Funding and conflicts of interest for this study.

15. If participants have any questions regarding the study.

16. Contact details of the person in charge: the principal investigators of the collaborating institutions and the research secretariat.

I have been explained the above items regarding this research.

Date of explanation: month/day/year

Name of the person providing the explanation: (self-attribution)

I have been fully informed by the person in charge of the study about its content. I understand the contents of the research and agree to participate.

Date of consent: month/day/year

Name: (self-attribution)
